# Utilization of Infrared Thermography in Assessing Thermal Responses of Farm Animals under Heat Stress

**DOI:** 10.3390/ani14040616

**Published:** 2024-02-14

**Authors:** Marcelo Daniel Ghezzi, Fabio Napolitano, Alejandro Casas-Alvarado, Ismael Hernández-Ávalos, Adriana Domínguez-Oliva, Adriana Olmos-Hernández, Alfredo M. F. Pereira

**Affiliations:** 1 Faculty of Veterinary Sciences, Veterinary Research Center (CIVETAN), Universidad Nacional del Centro de la Provincia de Buenos Aires (UNCPBA), CONICET-CICPBA, Tandil 7000, Argentina; 2 Scuola di Scienze Agrarie, Forestali, Alimentari ed Ambientali, Università degli Studi della Basilicata, 85100 Potenza, Italy; 3 Neurophysiology, Behavior and Animal Welfare Assessment, DPAA, Universidad Autónoma Metropolitana (UAM), Unidad Xochimilco, Mexico City 04960, Mexico; 4 Facultad de Estudios Superiores Cuautitlán, Universidad Nacional Autónoma de México (UNAM), Cuautitlan Izcalli 54714, Mexico; 5 Division of Biotechnology—Bioterio and Experimental Surgery, Instituto Nacional de Rehabilitación-Luis Guillermo Ibarra (INR-LGII), Mexico City 14389, Mexico; 6 Mediterranean Institute for Agriculture, Environment and Development (MED), Institute for Advanced Studies and Research, Universidade de Évora, Pólo da Mitra, Ap. 94, 7006-554 Évora, Portugal

**Keywords:** heat stress, farm animals, thermoregulations, thermal imaging

## Abstract

**Simple Summary:**

Heat stress is an event that causes health alterations and decreases the productive performance of farm animals. Therefore, it is important to establish methods that can help to evaluate the thermal state of animals noninvasively. According to the physiological response of vasodilation, when the brain detects an increase in body temperature, infrared thermography can be used to detect these peripheral changes and predict heat stress. The present review aims to analyze the neurobiological response associated with heat stress and how thermal imaging in different thermal windows can be used to recognize heat stress in farmed ungulates.

**Abstract:**

Heat stress is a condition that can affect the health, performance, and welfare of farm animals. The perception of thermal stress leads to the activation of the autonomic nervous system to start a series of physiological and behavioral mechanisms to restore thermostability. One of these mechanisms is vasodilation of peripheral blood vessels to increase heat loss through the skin. Due to this aspect, infrared thermography has been suggested as a method to assess the thermal state of animals and predict rectal temperature values noninvasively. However, it is important to consider that predicting rectal temperature is challenging, and its association with IRT is not always a direct linear relationship. The present review aims to analyze the neurobiological response associated with heat stress and how thermal imaging in different thermal windows can be used to recognize heat stress in farmed ungulates.

## 1. Introduction

Maintaining the thermal comfort of animals is one of the top priorities inside food production facilities [1]. High ambient temperatures cause heat stress (HS), triggering thermoregulatory changes that include physiological and behavioral responses, such as covering themselves in mud or using shade to reduce body temperature [2,3,4]. Continuous exposure to direct solar radiation can lead livestock to develop HS. In this context, although HS and febrile conditions cause an increase in body temperature, fever is caused by pyrogenic substances such as cytokines that act on hypothalamic centers, while HS does not necessarily involve pyrogens. Among the physiological changes that promote heat elimination, sweating and vasodilation of dermal blood vessels increase heat exchange with the environment and decrease body core temperature [5,6]. HS also causes alterations in the health and productive performance of farm animals, which is a threat to their welfare and the demand for animal food products [7]. Nonetheless, although these modifications aim to recover thermostability (a term used to define a state where heat production and loss are under balance), chronic exposure to HS has negative effects on animals’ health and productive performance.

These alterations are associated with the activation of stress-mediated pathways, such as the hypothalamic–pituitary–adrenal (HPA) axis and its connection to peripheral thermoreceptors that identify thermal variations [8,9]. The HPA axis facilitates the availability of energy resources and increases body heat production [10]. This effect leads animals to reduce feed intake and, consequently, daily weight gain (DWG) and milk yield [11,12]. These examples show the importance of recognizing and proposing strategies to monitor and maintain the thermal neutrality of farm animals.

One of the suggested tools to evaluate the thermal stability of animals is infrared thermography (IRT). Our previous studies have shown that IRT has benefits over the gold standard of rectal temperature (RT) since it is a noninvasive tool that evaluates peripheral vasomotor changes closely related to body core temperature when the animal is in a thermal neutral state and nondiseased [13]. Several studies have reported that increases in surface temperature can be related to increases in core temperature due to vasodilation of dermal blood vessels [14,15]. For example, zones with rich arteriovenous anastomoses, glabrous skin, or sparse hair are called “thermal windows” because these areas promote and facilitate heat exchange through vasomotor modulation [16], providing an approximation of body core temperature [17]. Particularly, ocular surface temperature is one of the most used regions to perform thermal imaging because it meets the previous characteristics and is also not affected by body size [18,19]. Eye surface temperature has shown positive and strong correlations with RT in healthy cats (r = 0.93) [20], and Bland–Altman testing has shown agreement between ocular temperature and RT in horses undergoing exercise and sheep during shearing [21,22]. However, no correlation between both temperatures was found in anesthetized mongrel dogs, probably due to the effect of anesthesia [19]. Therefore, thermal imaging and its interpretation highly depend on the characteristics of the evaluated body region, and currently, there is no consensus on the most practical thermal window to evaluate thermostability in domestic species. For this reason, this review aims to analyze the neurobiological response associated with thermal stress, the application of IRT, and the current thermal windows that have been used in farmed ungulates to recognize HS.

## 2. Neurobiological Response to Heat Stress

One of the current challenges for farms is maintaining the animals’ thermostability without affecting the productive performance of the species. The main reason why HS is associated with decreased productive performance is the physiological consequences on animals. These physiological consequences also differ among species due to the species-specific characteristics [23]. An example is the water buffalo and *Bos taurus*/*indicus* bovines, both ruminants with notable differences. On one hand, the water buffalo’s skin is thicker (6.5 mm) and has a smaller number of hair follicles (between 135 and 145 hair follicles/cm^2^), while conventional cattle have an average skin thickness of 4.3 mm and 3000 hair follicles/cm^2^ [24]. Furthermore, water buffaloes have a small number of functional sweat glands (394 vs. 2633 number/cm^2^) and dark skin, making them susceptible to heat retention [24,25,26]. Therefore, the susceptibility of animals to HS also depends on the species and even the breed.

To understand the effect of HS on the neurobiological response of animals to maintain their body core temperature within the thermoneutrality zone, it is necessary to define the meaning of thermoneutrality. According to Taylor [27] and Aggarwal and Upadhyay [28], this term refers to the range of environmental temperatures where animals do not require additional energy use to maintain body core temperature and where the physiological cost is minimal while productivity is maintained. Moreover, thermal neutrality can change depending on the temperature conditioning an animal is exposed to [29]. In the case of sheep, thermoneutrality is achieved within 12–32 °C [30,31]. In buffaloes, it is 13–24 °C [32,33], similar to cattle at 25 °C [34], and for most farm animals, it is considered to be between 4 and 25 °C [35]. A concept derived from the understanding of thermoneutrality zone is upper critical temperature. This refers to a point where the maximum body temperature that can be tolerated by an organism is reached (around 30–45 °C in mammals) [2,36]. This level of tolerance can be lower/higher according to the anatomical and physiological traits of animals. When this upper limit is exceeded, thermoregulatory mechanisms become ineffective and animals are not able to dissipate the excess heat, culminating in HS [37,38].

**Figure 1:**
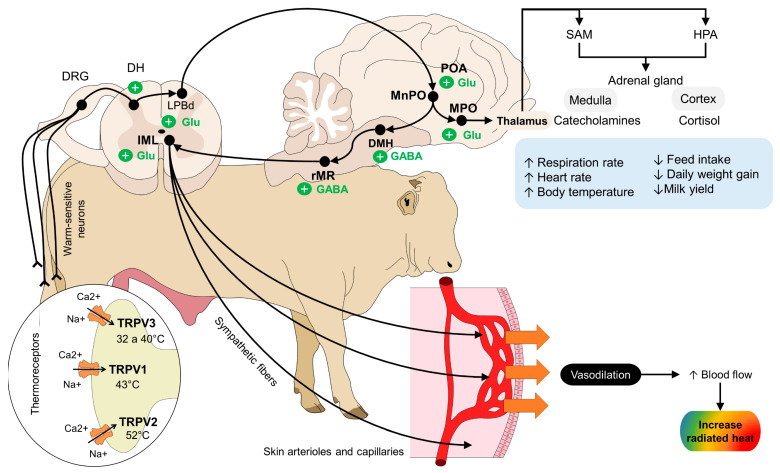
Neurophysiology of mammals under heat stress. The perception of noxious and non-noxious heat by peripheral thermoreceptors (TRP) triggers a series of electrical signals aiming to thermoregulate. Temperatures between 32 and 52 °C activate TRPV3, TRPV1, and TRPV2 ion channels. Warm-sensitive neurons transmit the thermal signaling into the DH, where the neurons synapse with the LPBd nucleus to start a physiological response against heat stress. After reaching the main thermoregulatory center (the POA, MnPO, and MPO), projections to the DMH, the rMR, and the IML activate the sympathetic fibers that innervate the skin arterioles and capillaries. The compensatory vasodilation response increases blood flow to, consequently, increase the amount of radiated heat through the skin to decrease body core temperature. Simultaneously, the connection of the MPO with the thalamus and sympathetic-mediated axis (SAM and HPA) causes the release of catecholamines and cortisol, which highly influence the physiological and behavioral mechanisms of thermoregulation in animals. DH: dorsal horn of the spinal cord; DMH: dorsomedial hypothalamus; DRG: dorsal root ganglion; HPA: hypothalamic–pituitary–adrenal axis; IML: intermediolateral laminae; LPBd: dorsal part of the parabrachial nucleus; MPOa: medial preoptic area; MnPO: median preoptic area; POA: preoptic area; rMR: rostral medullary raphe; SAM: symptom-adrenomedullary axis; TRP: transient receptor potential.

The recognition of these thermal changes (both environmental temperature and rise in body core temperature as a response to HS) is performed through thermoreceptors located in the skin, as mentioned by our research team [39]. These receptors, called transient receptor potential (TRP) channels, transduce thermal information into an electrical signal that is transmitted to the dorsal horn of the spinal cord [40,41]. Each of these TRPs is activated according to the temperature degree. For example, TRPV1, TRPV2, and TRPV3 subunits respond to temperature above 43, 52, and 33 °C, respectively [42,43]. The activation of these receptors initiates the physiological response of animals to HS. The integration of thermal signaling also activates other cerebral regions, such as the lateral parabrachial nucleus of the pons [44], where neurons synapse in other areas, such as the preoptic area, the anterior hypothalamus, and the dorsomedial hypothalamus, to start the thermoregulatory pathways, as shown in Figure 1 [5,45,46].

**Figure 2:**
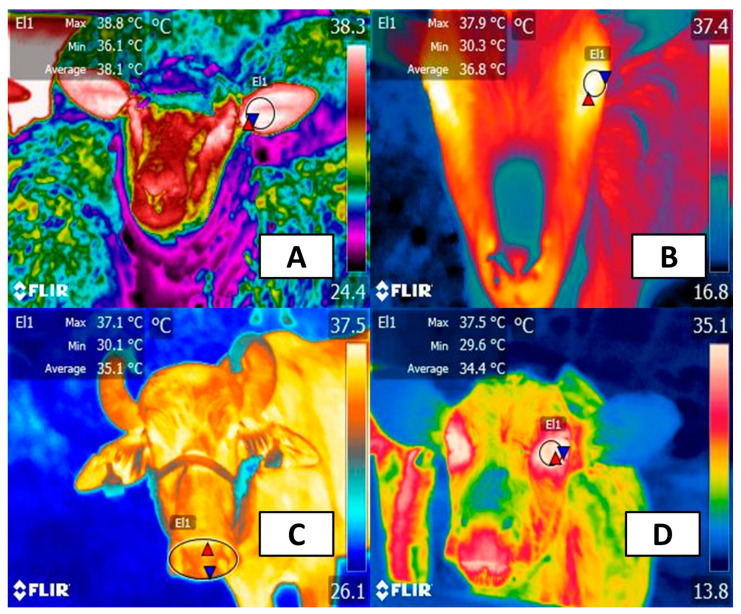
Suggested thermal windows in farm animals. (**A**) Auricular (El1). This window indirectly records the radiated temperature of the tympanic membrane, which is supplied by the auricularis medialis and the marginal arteries. (**B**) Ocular. This region considers the temperature coming from the eyeball and the eyelids (El1). Both are irrigated by the supraorbital vein and artery, the angular oculi vein, and branches of the ophthalmic artery. (**C**) Nasal. This window assesses the changes in the temperature of the exhaled air, also having irrigation by the superficial capillaries of the maxillary artery (El1). (**D**) Lacrimal caruncle. This region, like the ocular thermal window, reflects the circulation of the blood capillaries of the maxillary and infraorbital arteries, together with sympathetic innervation in the medial commissure of the eyelids (El1). Red triangles represent the maximum temperatures of the thermal windows. Blue triangles represent the minimum temperatures of the region of interest. FLIR thermal camera, model E60, lens FOL 18 mm, IR resolution 320 × 240.

Vasomotor response is one of the compensatory mechanisms used by most animals because modifying the diameter of the peripheral vessels closest to the skin dissipates heat by promoting a greater thermal exchange with the environment [47]. This was studied in buffaloes during transport by our team in Rodríguez-Gonzáles et al. [48], where IRT was used to evaluate the surface temperature in different body areas. We found an increase of up to 5 °C in the surface temperature of the pelvic limbs after transport. Likewise, the temperature of the parietal-frontal region increased by up to 10 °C after mobilization. These authors explain that the increase in surface temperature might be due to transport being a known stressor for farm animals. Moreover, this study showed that regions with a significant blood supply facilitate the elimination of heat to regulate body temperature. Something similar has been reported in camels, in whom increases in the surface temperature of the axilla, flanks, and hump were directly correlated with the rate of sweating due to high environmental temperature [49]. Other examples have been reported in pigs, in which sows exposed to noncooled pens (air temperature of 25.6 °C) had the highest temperature in the mammary gland (35.9 °C), a value that differed from that in sows kept under cooler pens (air temperature of 23.3 °C) [50]. These studies suggest that, for animals exposed to HS, vasomotor response is an important mechanism for heat elimination and that peripheral thermal response depends on the body region where it is evaluated [23,51]. Figure 2 shows the thermal response obtained by the authors of the present review from healthy farm species.

The vasomotor mechanism started from the perception of environmental temperatures is integrated in hypothalamic brain centers, such as the lateral parabrachial nucleus (LPB) [52]. From the LPB, depending on the stimulus, neurons in the dorsal subnucleus of the LPB are activated during HS [53]. The information on these neurons is projected to the median preoptic nucleus (MnPO) modulates’ heat loss by exciting or inhibiting neurons in other structures, such as the rostral magnus raphe, which transmits the signal to the efferent sympathetic preganglionic fibers of the intermediolateral (IML) columns of the spinal cord that innervate and provide vasomotor control to cutaneous blood vessels and brown adipose tissue (BAT) [54,55].

**Figure 3:**
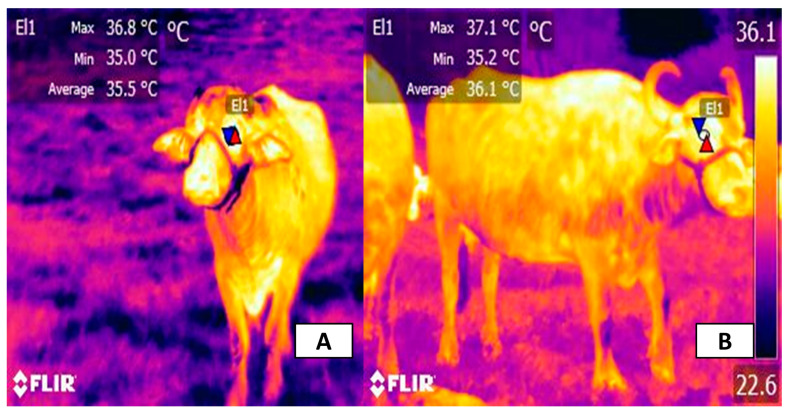
Effect of solar radiation on the surface temperature of the periocular region of water buffaloes. (**A**) Morning eye temperature. The surface temperature of the periocular region (El1) in the morning has a maximum value of 36.8 °C, while the average and minimum temperatures are 35.5 and 35 °C. (**B**) Eye temperature at midday. In comparison with the temperature shown in the morning, the maximum temperature increased by 0.3 °C, the average temperature increased by 0.2 °C, and the minimum temperature decreased by 0.6 °C (El1), which is possibly due to the solar radiation during the time of the day, where animals are most susceptible to HS. Red triangles represent the maximum temperatures of the thermal windows. Blue triangles represent the minimum temperatures of the region of interest. FLIR thermal camera, model E60, lens FOL 18 mm, IR resolution 320 × 240.

Due to the sympathetic cutaneous innervation, two fundamental thermoregulatory responses can be observed: vasodilation (by cholinergic fibers) and vasoconstriction (by noradrenergic fibers) [5,56]. Therefore, when animals are exposed to high ambient temperatures, vasodilation of the capillaries increases blood flow at the superficial level to increase passive heat dissipation through the skin [55]. This effect is more prominent in those regions with rich subdermal capillaries and with a lower density of hair or glabrous skin, zones that facilitate thermal exchange (also called thermal windows) [23,57]. Direct exposure to solar radiation when animals remain in unshaded areas significantly increases the surface temperature of animals as an effort to decrease heat load. This was reported in Santa Ines sheep and their crosses with Dorper sheep maintained under direct solar radiation for 8 h [58]. Both rectal and ocular temperature increased by up to 39.23 and 39 °C, respectively, reaching an average of 1.54 °C above initial temperatures. This effect is also shown in Figure 3, where the effect of solar radiation on the ocular surface temperature in healthy water buffaloes was assessed by the authors of the present review.

## 3. Physiological Responses to Mitigate Heat Stress

Apart from these vasomotor changes, Gupta and Mondal [59] reported that thermal imbalance due to HS causes a consequent HPA-induced release of cortisol and adrenocorticotropic hormone (ACTH), as well as catecholamines. These substances cause physiological and metabolic alterations. On one hand, catecholamines start the “flight-and-fight” response that is often accompanied with tachypnea, tachycardia, and increases in body core temperature, while cortisol promotes catecholamine release, mobilization of energy, and immunosuppression when released chronically [60,61]. For example, sheep chronically exposed to HS (THI 27.9) had higher hair cortisol levels when deprived of water for 3 h (2.78 pg/mg) than those animals without water restriction (2.63 pg/mg) due to the endocrine response to the stressor [62].

Catecholamines not only act on the cardiorespiratory system, but also have effects on other sympathetic-mediated changes, such as pupillary diameter. Recently, pupillometry has been used to assess the response of animals exposed to different stressors, including thermal stress. In this context, Lopes Neto et al. [63] evaluated pupillary dilation response as an indicator of thermal stress in six Boer crossbred goats. The findings showed that an increase of 26.96 mm2 in the pupillary area was related to heart rate (HR) increasing by 16 beats per minute (bpm) and respiratory rate (RR) increasing by 20 breaths per minute when the air temperature was 33 °C. The authors explain that HS triggers sympathetic changes such as tachycardia and tachypnea to increase blood flow circulation and heat dissipation by evaporative heat loss. A similar study was performed by Marques et al. [64], who evaluated the effect of exposure to hot air at different temperatures (26, 29, and 33 °C) in crossbred goats of the Boer breed (^3^/_4_ Boer and ¼ no defined racial pattern). A significant increase in HR and RR of up to 50% was recorded. Similarly, there was a significant decrease of 0.65 kg in daily weight gain (DWG) when exposed to the hottest air temperature (33 °C). Furthermore, Yamin et al. [65] evaluated the association of THI with physiological and biochemical responses in Malabari crossbred and Attappady black goats. They found positive correlations between the increase in THI and HR (r = 0.29) and RR (0.23). Additionally, they observed that in the animals of the Malabari breed, blood pH decreased by 0.08 and CO_2_ increased by 2 mmHg, in contrast with the animals of the Attappady black breed. For these authors, this shows that there is a difference between breeds in their capacity for habituation to HS.

Although these changes aim to maintain homeostasis by activating several compensating mechanisms (e.g., vasomotor changes and increases in evaporative heat loss), they also have consequences when animals are chronically exposed to HS. In this context, changes in respiratory pattern and at the vasomotor level can affect the animal’s acid–base balance. Due to the vasodilation of blood capillaries, gas exchange becomes difficult in peripheral tissues by decreasing CO_2_ elimination despite the increase in RR [66]. This is possibly due to the affinity of oxygen to hemoglobin, which can cause difficulty in the release of this molecule, decreasing blood pH and activating buffer systems to restore homeostasis after HS [67,68].

Farm animals also adopt several thermoregulatory behaviors to increase heat dissipation by radiation, convection, or evaporation to cool themselves [5,48]. Shade seeking, changes in body posture (standing or lying time), reduced feed intake, wallowing, water intake, reduced activity, and urinating frequently are among the most frequent behavioral responses that livestock use to respond to HS [69,70]. For example, shade seeking is one of the first behavioral responses of farm animals because it has been shown to reduce radiant heat load in cattle by about 45% [71]. Species like sheep have been reported to have a preference for shaded areas of approximately 56–43% [72], and the provision of shade in the same species effectively reduces the compensatory tachypnea that sheep use to maintain thermostability [73]. Nonetheless, another study performed by our research team [74] found that water buffaloes resort to wallowing in mud or water to decrease their body core temperature.

In the case of pigs, Bonneau et al. [75] reported an increase in the amount of time the animals were lying down on the side—to dissipate heat by contact with cool surfaces—by up to 5.9% when the temperature increased by 1 °C. The authors also determined that creole pigs (dark skin) spent more time lying down than large white pigs (lighter skin) (4%), probably due to the anatomical differences between breeds, which could help to identify heat-tolerant breeds for certain pig production units where ambient temperatures are high. In small-tail Han sheep raised indoors without climate control, Li et al. [76] observed that ewe and ram lying behavior increased according to the THI increase (up to 45.10 ± 2.42%). Nonetheless, ewes seem to have a higher tolerance to HS than ram based on fewer incidences of standing (23.94 ± 1.98% vs. 38.42 ± 2.25%) and lying (11.56 ± 3.28% vs. 44.38 ± 10.60%) during extremely severe HS. Likewise, goat and sheep resort to other behaviors such as increasing the frequency of water intake or shade seeking to mitigate the effects of HS [77]. Consequently, HS triggers a cascade of physiological and behavioral responses to decrease body core temperature and restore thermostability, and the degree of the said response is highly influenced by the species.

The aforementioned physiological responses and the activation of the HPA axis are addressed in the case of heat stress. Nonetheless, this stress response is not unique to thermal stress. It can be triggered by other stressors inherent in livestock farming, such as transport, social isolation, or having diseases [8,9,48]. This could be a potential limitation of using IRT to detect heat stress, since IRT may capture not only thermal stress but the generalized stress response. Thus, the combination of physiological and behavioral measurements in combination with IRT is suggested as the best alternative for thermoregulatory imbalance detection.

## 4. Productive and Physiological Consequences of Heat Stress

Thermoregulatory changes to mitigate HS aim to maintain body core temperature within acceptable ranges according to the species [78]. Since HS can be considered a stressor that challenges the homeostasis of animals, it has several consequences that might alter the health and fitness of animals.

Regarding the effects on the productive performance of animals, it has been reported that HS decreases DWG. For example, goats exposed to HS have lower plasma glucose and cholesterol levels [79]. This might be due to a reduction in digestibility, utilization efficiency, and feed consumption and the role of catabolic hormones that are present during HS [80,81]. Moreover, decreases in glucose levels might be related to a decrease in nutrient availability when animals reduce their feeding intake, while cholesterol levels can decrease as a response to low acetate concentration or increased body water [79]. Reduced absorption from nutrient digestion also causes a lower growth rate. According to a comprehensive review by Lima et al. [82], although goats are considered a resilient species to HS when chronically exposed, their energy metabolism is affected. The activation of the HPA axis facilitates the availability of energy resources due to the secretion of cortisol, which allows the increase in body temperature. Therefore, this increase in temperature limits feed consumption and growth rate [83]. This coincides with what was observed by Pragna et al. [84] in 36 goats from three native breeds, where the level of growth performance during HS in the summer was evaluated. The results showed a reduction of 10%, 8%, and 6% in growth for the Osmanabadi, Malabari, and Salem Black breeds, respectively, suggesting that thermal stress has a direct impact on the productive level of the animal [85].

In addition to DWG alterations, decreases in milk yield have also been reported [86,87,88,89]. This is related to the reduction in feed consumption, which can negatively affect energy balance, leading to a decrease in milk synthesis and ejection. According to Berman [90], maintaining dairy cattle under ambient temperatures above 35 °C activates the stress-related systems and reduces milk yield. Interestingly, the reduction in feed consumption increases the energy maintenance requirements of dairy cattle by 30% [91].

Likewise, a comparative study conducted by Gaafar et al. [92] in Holstein dairy cattle found that an increase in THI reduced total milk production by 39% in the summer, in contrast with a reduction of 31% in the spring and 29% in the winter. Joksimovic-Todorovic et al. [93] also found that the average total milk yield was 3 L higher in the spring compared with the production during the summer due to a lower food intake and alterations in the absorption of carbohydrates, lipids, and proteins [94]. Therefore, HS has a direct Impact on both the physiological and productive functions of animals, causing acid–base imbalances or a decrease in DWG, feed intake, or milk yield. Therefore, these consequences must be considered in management strategies to reduce the effects of HS on livestock.

## 5. Infrared Thermography as a Tool to Assess Thermostability in Farm Animals

IRT has been suggested as a tool to evaluate the thermal stability of animals under HS due to peripheral vasomotor changes [95]. Vasodilation increases blood flow to dissipate heat. This also increases the amount of radiated heat from the skin. Due to this, IRT can detect these vasomotor changes [96,97]. A study carried out by Marques et al. [14] evaluated the potential of IRT as a tool to assess thermal changes in six male Boer crossbred goats exposed to three different temperatures of 26, 30, and 34 °C. By assessing the surface temperature of the ocular region, head, shoulder, and hindlimbs, the authors observed that the surface temperature of all regions significantly increased by 2 °C after exposure to the highest temperature. Moreover, it was also observed that the ocular temperature was positively correlated with the rectal temperature of the animals (r = 0.95). Similarly, in 24 Blanca Serrana goats where surface eye temperature was evaluated, increases in IRT response (+1.1 °C) and in RR (+20 breaths per minute) were observed after exposure to hot air, which were positively correlated with each other (r = 0.56) [15]. These results coincide with what was found in domestic cattle by Peng et al. [98], who assessed the relation between rectal temperature and surface temperature under different THIs in 488 Holstein dairy cows. The authors observed that an increase in THI significantly increased the surface temperature of the eye, ear, cheek, forehead, flank, rump, fore udder, and rear udder. In particular, the forehead was considered the most suitable region to detect changes in body core temperature with a THI of 66.8–71.4. Furthermore, they found a significant correlation between rectal temperature and surface temperature (r = 0.55).

These studies show the association between surface thermal response and rectal temperature due to hyperthermia caused by stress.

### Thermal Windows Used to Assess Thermostability of Animals

Scientific evidence indicates that IRT is a useful tool to establish the thermal status of animals and its relation to other physiological parameters associated with HS [13]. However, it is essential to establish which thermal window could be more suitable to show sympathetic activity after HS [99,100]. One of the most used thermal windows to find associations with rectal temperature is the ocular region [14,15,98]. This anatomical region, in particular, the lacrimal caruncle blood supply from the infraorbital artery, is a region highly sensible to vasomotor changes [9]. Moreover, this artery is related to sympathetic adrenergic fibers, so an increase in ocular temperature might be a predictor of a stress response.

Mincu et al. [101] found that surface temperature also changes when exposed to other psychological stressors. In dairy Holstein-Friesian cows, an increase of 2 °C in ocular surface temperature was reported after social isolation. Similarly, a study performed in water buffaloes showed that increases in eye temperature might be related to transport stress. This coincides with what was reported by Stewart et al. [102], who evaluated six Holstein cattle under six different random treatments (control, ACTH, corticotropin-releasing factor at 20 or 40 μg, epinephrine, and social isolation). The authors found that cortisol increases were accompanied by a significant increase of 0.3 °C in eye temperature during catheterization and with the administration of epinephrine (0.5 °C). In contrast, the administration of ACTH and CRH did not influence thermal imaging despite the increased level of cortisol. Due to these results, some other authors have concluded that ocular temperature can indirectly assess the response of animals subjected to stress and even pain [103,104,105].

Another thermal window that has been suggested is the nasal region because the heat eliminated in this region is related to RR. In this context, Lowe et al. [106] evaluated the thermal fluctuations of the nasal region in 5 Hereford calves of 27 days. It was observed that the nasal thermal window had a high correlation with RR evaluated through flank movements (r = 0.93). This coincides with what was reported in a study performed on 22 cows (Friesian and Friesian x Jersey breed) exposed to a startle. In these animals, RR assessed with the technique of the study above recorded an average difference of −0.01 ± 0.87. Moreover, the thermal response of the nasal region reliably predicted the heart rate variability due to changes in respiratory rate [107]. These results suggest that the nasal region is useful for evaluating RR, and in animals exposed to HS, RR tends to increase (+4.3 breaths per minute) as a mechanism to dissipate up to 30% of heat production [108,109].

Ear surface temperature has also been assessed to monitor body temperature in livestock. In cows and calves, rectal and vaginal temperatures were compared with the surface temperature of the back of the ear, eye, shoulder, and vulva [110]. The results showed that ocular and ear maximum temperature were suitable regions to assess body temperature. Other thermal windows, such as the back and flank regions, have also been suggested to evaluate animals’ thermoregulation. An example of this was reported by Sevegnani et al. [111], who evaluated the thermoregulatory response of 15 daily Murrah buffaloes during the premilking and postmilking periods. The surface temperatures of the ear, neck, and back increased between 5 and 12 °C during postmilking. Nonetheless, the authors discussed that the thermal response of regions such as the flanks and neck is highly influenced by environmental conditions such as THI and air temperature. Therefore, the response on these windows might not be directly related to the stressor per se. An alternative is the assessment of pelvic or thoracic limbs, where vasodilation has been reported in animals undergoing HS [23]. Likewise, another possible thermal window is the frontal-parietal region, which is possibly associated with the circulation present in the horns and might serve as a region to dissipate heat [112]. Theusme et al. [113] reported that the anatomical region with the highest correlation with rectal temperature was the forehead (r = 0.58) in Holstein heifers during the summer.

Consequently, the ocular and nasal thermal windows are suggested to be the most acceptable regions to recognize and evaluate thermal stress in animals. Nonetheless, it would be suitable to consider other regions such as the horns or limbs that are also related to peripheral thermoregulatory mechanisms and might give an appreciation to HS.

## 6. Perspectives

The neurobiological response associated with thermoregulation indicates the importance of free nerve endings present in the skin. In this context, a possible area of further research could aim to compare if, between species, there are differences regarding TRP activation threshold or the presence/absence of certain thermoreceptors. Likewise, another area of research is to apply IRT in farms where the use of natural or artificial shadows is being implemented. This might reduce the influence that direct solar radiation has on IRT readings, as shown in agroforest systems [114]. Comparisons between farms or between animals from the same unit could help to establish the benefits of these additions to promote thermostability in animals.

Finally, one aspect that needs emphasis is that IRT is suggested as a complementary tool to other current technologies and physiological/behavioral evaluations due to its limitations. For example, Unruh et al. [115] did not find an association between increases in surface temperature (+2 °C) and panting score in crossbred beef heifers subjected to thermal stress. This lack of association between rectal temperature and other physiological parameters suggests that IRT might have some limitations that need to be considered when applying thermal imaging to assess the thermal state of animals.

The possible limitations of IRT are related to the factors that can affect the evaluation of the thermal response. For example, ocular surface temperature has shown to be highly affected by environmental temperature [116,117,118]. Moreover, apart from external factors, individual characteristics such as the presence/absence of hair or coat length/color/type also influence the amount of heat loss/gain [17]. In this regard, the length and hair color can cause a difference in temperature of up to 2 °C [119,120,121]. Another factor is wind speed. In the case of dairy cattle, it is reported that a wind speed of 7 km/h can reduce ocular surface temperature by 0.43 ± 0.13 °C, while a speed of 12 km/h can alter the temperature by 0.78 ± 0.33 °C [122]. Although this tool has been shown to have a direct relationship with physiological parameters such as rectal temperature, HR, and RR, the selection of the thermal window needs to be validated and the different factors that can alter IRT must be considered to precisely assess HS [123].

## 7. Conclusions

Heat stress in farm animals is a condition where high ambient temperatures exceed the thermostability wherein they can maintain a balance between produced and dissipated heat. When animals are exposed to HS, peripheral vasomotor changes increase heat loss, together with other physiological responses, such as tachypnea and tachycardia. Due to this vasomotor mechanism, IRT can be used as a method to identify changes in body surface temperature due to vasodilation. Currently, ocular surface temperature is considered the region that is highly correlated with body core temperature. However, adopting other thermal windows requires additional research to determine the differences between species and under different temperatures to establish a window that accurately evaluates the thermal state of animals.
